# Comparative Molecular Similarity Indices Analysis of 1-(Naphthylalky1)-1H-imidazole Analogs with Antiepileptic Activity

**DOI:** 10.4103/0975-1483.71635

**Published:** 2010

**Authors:** S Ganguly, R Mishra

**Affiliations:** *Department of Pharmaceutical Sciences, Birla Institute of Technology, Mesra, Ranchi - 835 215, Jharkhand, India*

**Keywords:** 3D QSAR, antiepileptic agents, CoMSIA, epilepsy, imidazole

## Abstract

A three-dimensional quantitative structure-activity relationship (3D QSAR) of 44 structurally and functionally diverse series of 1- (Naphthylalkylimidazoles) as antiepileptic agents was studied using the Comparative molecular similarity indices analysis (CoMSIA) method. A training set containing 34 molecules served to establish the models. The optimum CoMSIA model obtained for the training set were all statistically significant, with cross-validated coefficients (q^2^) of 0.725 and conventional coefficients (r^2^_ncv_) of 0.998. The predictive capacities of the model were successfully validated by using a test set of 10 molecules that were not included in the training set. CoMSIA model (Model 1) obtained from the hydrophobic and Hbond acceptor field was found to have the best predictivity, with a predictive correlation coefficient (r^2^_pred_) of 0.67. The information obtained from this 3D-QSAR model can be used to guide the development of imidazoles as novel antiepileptic agents.

## INTRODUCTION

Epilepsy is a chronic and often progressive disorder characterized by the periodic and unpredictable occurrence of epileptic seizures, which are caused by an abnormal discharge of cerebral neurons.[1] There is a continuing demand for new anticonvulsant agents, as it has not been possible to control every kind of seizure with the currently available antiepileptic drugs. Moreover, the current therapy of epilepsy, with modern antiepileptic drugs, is associated with dose-related side effects, chronic toxicity, and teratogenic effects.[2,3] Therefore, new antiepileptic drug development, with approved therapeutic properties, is an important challenge for medicinal chemists.

**Figure 1:**
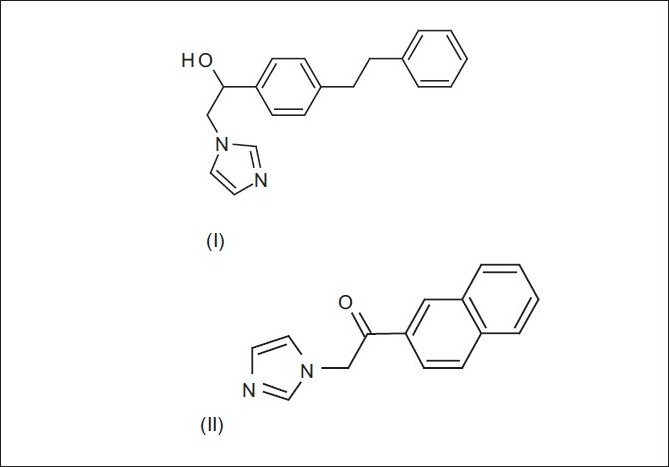
Chemical structures of Denzimol (I) and Nafimidone (II)

Hydantoin was used as an antiepileptic from the 1860s, but the utilization of substituted imidazoles in the treatment of epilepsy was brought in with the synthesis of Denzimol (I)[4] and Nafimidone (II),[5] respectively [Figure 1]. A sound understanding of the structural requirements of imidazoles for antiepileptic activity is important in guiding and optimizing the drug design efforts.

Comparative molecular field analysis (CoMFA) and Comparative molecular similarity indices analysis (CoMSIA) are powerful tools to build and design an activity model (QSAR), for a given set of molecules in a rational drug design and related applications.[6,7] The CoMFA methodology is based on the assumption that the changes in the biological activity correlate with the changes in the steric and electrostatic fields of the molecules.[8] The CoMSIA[8,9] method was introduced by Klebe and includes additional molecular fields, such as, the lipophillic and hydrogen bond potential. CoMSIA introduces the Gaussian function for the distance dependence between the molecular atoms and the probe atoms, in order to avoid some of the inherent deficiencies arising from the Lennard-Jones and Coulomb potential functional forms. In CoMSIA, five different similarity fields, namely, steric, electrostatic, hydrophobic, hydrogen bond donor, and hydrogen bond acceptor fields were calculated. These fields were selected to cover the major contributions to ligand binding and have several advantages over CoMFA, such as, development of a more robust 3D QSAR model, with no arbitrary cut-offs and more intuitively interpretable contour maps. This article describes the Comparative Molecular Similarity Indices Analysis (CoMSIA) of a series of 1-(Naphthylalky1)-1H-imidazole analogs [Table 1]. On the basis of the CoMSIA model generated by us we attempted to elucidate a structure activity relationship to provide useful information for the design of more selective and potent antiepileptic imidazole analogs.

**Table 1:** Dataset used for CoMSIA analyses

Compound no.	Substituent				ED_50_^a^ (mg/kg)
	A	R	Napthyl	N	
1	O	-	2	1	15
2	O	6-Cl	2	1	51
3	O	6-CH_3_	2	1	24
4	O	6-C_2_H_5_	2	1	12
5	O	6,7-(CH_3_)_2_	2	1	25
6	O	6-OCH_3_	2	1	31
7	O	1-OCH_3_	2	1	23
8	O	7-CH_3_	1	1	22
9	O	7-C_2_H_5_	1	1	13
10	O	6,7-(OCH_3_)_2_	1	1	79
11	OCH_2_CH_2_O	-	2	1	12
12	OCH_2_C(CH_3_)_2_ CH2O	-	2	1	26
13	(OCH_3_)_2_	-	2	1	40
14	S(CH_2_)_3_S	-	2	1	65
15	SCH_2_CH_2_S	-	1	1	26
16	(SCH_3_)_2_	-	2	1	32
17	(SC_2_H_5_)_2_	-	2	1	35
18	(S-i-C_3_H_7_)_2_	-	2	1	86
19	(SC_6_H_5_)_2_	-	2	1	60
20	(SCH_2_C_6_H_5_)_2_	-	2	1	100
21	OCH_2_CH_2_O	-	2	2	35
22	H_2_	-	2	1	22
23	H_2_	-	2	0	25
24	OH	-	2	1	74
25	OH	-	1	1	34
26	OCH_3_	-	2	1	10
27	OC_2_H_5_	-	2	1	11
28	OCH_3_	-	1	1	19
29	p-OC_6_H_4_CI	-	2	1	46
30	o-OC_6_H_4_CH_3_	-	2	1	33
31	OCOC_2_H_5_	-	2	1	23
32	SCH_3_	-	2	1	37
33	OH	-	2	2	13
34	OCH_3_	-	2	2	11
35*	O	4-CH(CH_3_)_2_	1	1	28
36*	O(CH_2_)_3_O	-	2	1	17
37*	OCH(CH3) (CH_2_)_2_O	-	2	1	19
38*	OCH_2_CH2O	-	1	1	19
39*	SCH_2_CH_2_S	-	2	1	26
40*	(S-n-C_3_H_7_)_2_	-	2	1	100
41*	(S-i-C_4_H_9_)_2_	-	2	1	100
42*	O	-	2	2	10
43*	O-n-C_4_H_9_	-	1	1	16
44*	OCOC_6_H_5_	-	2	1	19

* Test set molecules (25%)

a ED_50_ is Median Effective Concentration

## MATERIALS AND METHODS

### Data set

Reported data on a series of 44 1-(Naphthylalky1)-1H -imidazole derivatives[5] were used [Table 1]. The ED_50_ data for a Maximal Electroshock induced seizure (MES test) were used for QSAR analysis, as a dependent parameter, after converting the reciprocal of the logarithm of ED_50_ (p ED_50_) values. ED_50_ was the dose of a drug that was pharmacologically effective for 50% of the population exposed to the drug. The total set of the imidazole analogs were segregated into the training set and the test set, in an approximately 4:1 ratio, each containing 34 and 10 molecules, respectively. The division was made at random with bias given to structural diversity in both the training set and the test set.

### Molecular modeling

The CoMSIA studies reported herein were performed using SYBYL 7.1[10] molecular modeling software installed on a Dell computer, with Red Hat Linux Enterprise Version 3.0 (with 512 MB of memory). All the compounds were built from fragments in the SYBYL database. Each structure was fully geometry-optimized using the standard Tripos force field with a distance-dependent dielectric function, until a Root Mean Square (RMS) deviation of 0.001 kcal mol ^−1^ Å^−1^was achieved. All the compounds were then subjected to simulated dynamic annealing with 100 cycles. The least energy conformations were selected for each compound and further energy minimized using Powell (100 iterations) and Conjugation gradient (10,000 iterations) methods. Gasteiger–Huckel charges were computed for all molecules after energy minimization.

### Alignment

**Figure 2:**
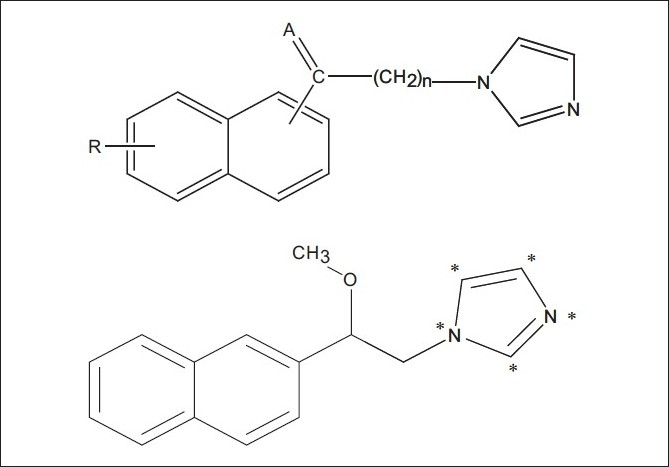
Template molecule 26 with common substructure labeled with *

**Figure 3:**
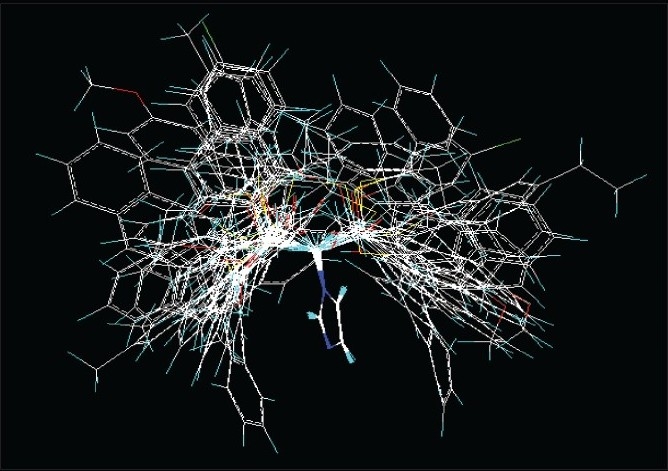
Alignment of training set molecules

Molecular conformation and orientation is one of the most sensitive input areas in 3D-QSAR studies. In the present study, superimposition of the molecules was carried out by DATABASE ALIGNMENT, using compound **26** [Table 1] as a template structure. The molecules were aligned to the template molecule by using a common substructure labeled with *, as shown in Figure 2. The aligned molecules are shown in Figure 3.

### CoMSIA interaction energy calculation[8]

In CoMSIA field energy calculation, the probe atom with radius 1 Å, charge + 1.0, hydrogen bond donating + 1.0, hydrogen bond accepting + 1.0, and hydropobicity + 1.0 were used. An attenuation factor of 0.3 was used to estimate the steric, electrostatic, hydrophobic, hydrogen bond donor, and acceptor fields in CoMSIA.

### Partial least square (PLS) analysis[11]

PLS regression technique is especially useful in common cases where the number of descriptors (independent variables) is comparable to or greater than the number of compounds (data points) and / or there exist other factors leading to correlations between variables. The column filtering value(s) were set to 2.0 kcal/mol, to improve the signal-to-noise ratio. Cross-validations were performed by the Leave-One-Out (LOO) procedure, to determine the optimum number of components (N). The cross-validated r^2^ that resulted in optimum number of components and lowest standard error of prediction were considered for further analysis. The final analysis was performed to calculate conventional r^2^ using the optimum number of components. Bootstrapping analysis for 100 runs was performed.

### Predictive correlation coefficient[8,12]

The predictive power of the model, predictive correlation coefficient (r^2^_pred_), based on molecules of the test set was calculated by the following equation.

$${r^{2}}_{\mathrm{pred}}\;=\;\left(\mathrm{SD}\;-\;\mathrm{PRESS}\right)/\mathrm{SD}$$

Where SD is the sum of the squared deviations between the biological activities of each molecule and the mean activity of the training set of molecules and PRESS is the sum of squared deviations between the predicted and actual activity values for every molecule in the test set.

## RESULTS

**Figure 4:**
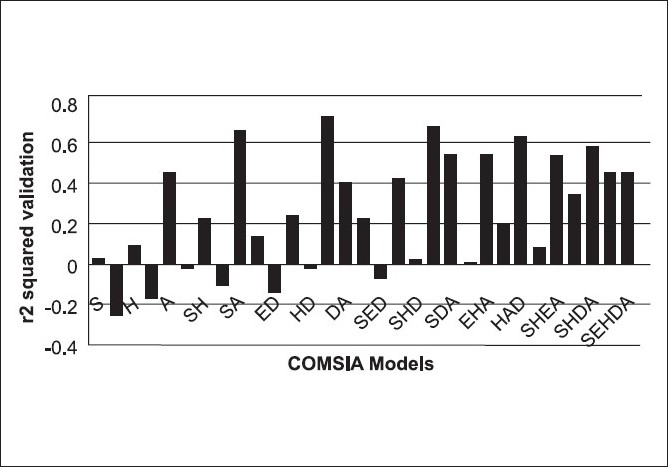
Plot of r^2^ cross-validated versus 31 different CoMSIA models

**Figure 5:**
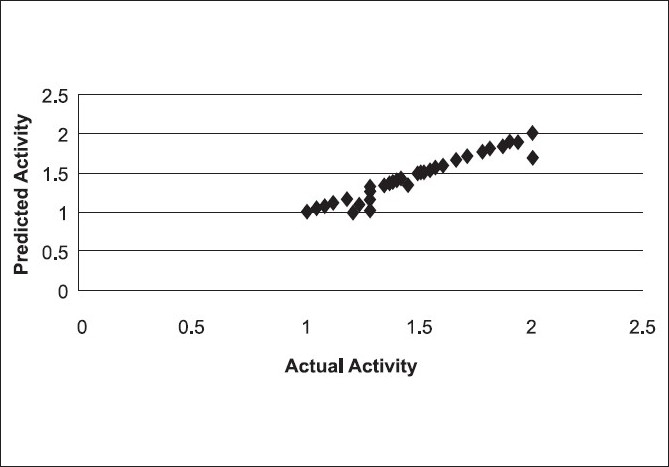
Plot of predicted versus actual pED_50_ values of molecules for CoMSIA Model 1 (HA)

**Figure 6:**
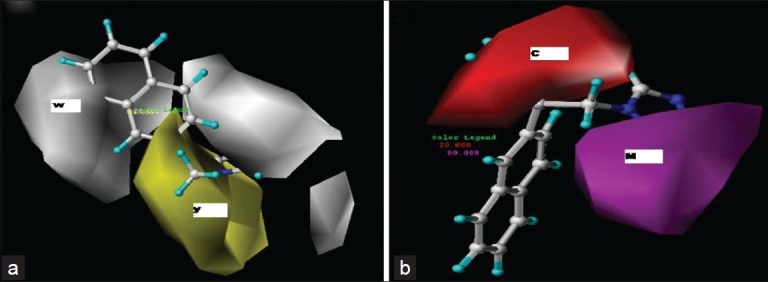
The CoMSIA hydrophobic (a) and hydrogen bond acceptor (b) contour maps. One of the most active molecules, 26, is shown in the background. Yellow (Y) is a hydrophobically favored region, white (W) hydrophobically disfavored region, magenta (M) color is a hydrogen acceptor favored region, cyan (C) color is a hydrogen acceptor disfavored region

Based on the predictive correlation coefficient (r^2^_pred_ = 0.67), the combination of hydrophobic and hydrogen bond acceptor fields in CoMSIA gave the best results (Model 1), giving a cross-validation correlation coefficient of 0.725 and a conventional correlation coefficient of 0.998. The other combinations such as (i) steric, hydrophobic, and hydrogen bond acceptor fields (Model 2) and (ii) steric and hydrogen bond acceptor fields (Model 3) in CoMSIA also gave statistically significant models. All other combinations in CoMSIA gave statistically insignificant results [Figure 4]. Model 1 of CoMSIA was used for final analysis and predictions. A high r^2^ value of 0.998 during 100 runs of bootstrapped analysis further supported the statistical validity of the model. The results of PLS analysis are shown in Table 2. A plot of predicted (CoMSIA) versus the actual activity for the training set molecules is shown in Figure 5. Figure 4 represents the plot of the cross-validated correlation coefficient versus all the CoMSIA models. The actual and predicted activity of training and test set for all CoMSIA models are given in Tables 3 and 4, respectively. The 3DQSAR contour maps revealing the contribution of the CoMSIA fields is shown in Figure 6. The contributions of the hydrophobic and hydrogen bond acceptor fields of CoMSIA are in the ratio 4:6 [Table 2].

**Table 2:** Statistics of CoMSIA models

Parameters	HA (Model 1)	CoMSIA	
		SHA (Model 2)	SA (Model 3)
r2cv	0.725	0.682	0.661
r2ncv	0.998	0.997	0.995
SEE	0.013	0.016	0.020
F	3111.632	1859.435	1235.770
r2bs	0.998	0.998	0.996
r2pred	0.6671	0.1351	−0.0981
Component	5	5	5
Fraction			
Steric	-	0.211	0.352
Electrostatic	-	-	-
Hydrophobic	0.438	0.311	-
Acceptor	0.562	0.459	0.648
Donor	-	-	-

r^2^_cv_: Cross-validated correlation coefficient, N: Number of components, r^2^: Conventional correlation coefficient, SEE: Standard error of estimate, PRESS: Predicted residual sum of squares of test set molecules, r^2^_pred_: Predictive correlation coefficient, r^2^_bs_: Correlation coefficient after 100 runs of bootstrapping analysis, S: Steric field, H: Hydrophobic field, A: hydrogen bond acceptor field

**Table 3:** Experimental and predicted activities of compounds in training set

Structure no.	Actual activity (pED_50_^b^)	CoMSIA (Predicted activity)		
		Model 1 (HA)	Model 2 (SHA)	Model 3 (SA)
1	1.17613	1.16640	1.17216	1.16571
2	1.70757	1.72051	1.70685	1.68225
3	1.38021	1.39070	1.40259	1.35321
4	1.07919	1.06169	1.08657	1.08432
5	1.39794	1.40956	1.38229	1.40084
6	1.49136	1.50355	1.49943	1.50450
7	1.36172	1.36496	1.38572	1.40760
8	1.34242	1.34513	1.36913	1.36773
9	1.11394	1.12009	1.11628	1.11148
10	1.89762	1.90523	1.90756	1.92671
11	1.07919	1.08055	1.07728	1.09711
12	1.41497	1.41269	1.39408	1.40669
13	1.60205	1.59645	1.58794	1.59678
14	1.81291	1.82170	1.81479	1.81102
15	1.41497	1.41112	1.42958	1.44960
16	1.50514	1.52417	1.53251	1.51736
17	1.54406	1.53367	1.52096	1.53887
18	1.93449	1.90724	1.91251	1.89186
19	1.77810	1.77306	1.77659	1.7852
20	2.00000	2.01645	2.01997	2.00159
21	1.54406	1.54296	1.53474	1.53512
22	1.34242	1.33465	1.33744	1.33597
23	1.39794	1.41050	1.40762	1.41176
24	1.86923	1.83959	1.84527	1.85428
25	1.53147	1.51507	1.50896	1.53133
26	1.00000	0.99129	0.99811	1.00204
27	1.04139	1.04901	1.03550	1.04202
28	1.27875	1.27785	1.29609	1.27773
29	1.66270	1.67162	1.67825	1.68669
30	1.51851	1.51225	1.51949	1.51104
31	1.36172	1.36580	1.34753	1.34561
32	1.56820	1.57128	1.56144	1.55484
33	1.11396	1.10643	1.11236	1.10198
34	1.04143	1.05256	1.02817	1.01496

pED_50_^b^ = −LogED_50_

**Table 4:** Experimental and predicted activities of compounds in test set

Structure no.	Actual activity (pED_50_^b^)	CoMSIA (Predicted activity)		
		Model 1 (HA)	Model 2 (SHA)	Model 3 (SA)
35*	1.44710	1.35280	1.23811	1.44710
36*	1.23045	1.09328	1.11691	0.90422
37*	1.27875	1.04060	1.00268	0.98745
38*	1.27875	1.15136	1.04132	0.87593
39*	1.41497	1.44695	1.30756	1.27258
40*	2.00000	1.69197	1.42497	1.47527
41*	2.00000	1.70970	1.48629	1.39003
42*	1.00000	0.99926	1.15160	1.17321
43*	1.20411	0.984424	1.20258	1.03100
44*	1.27875	1.32600	1.48527	1.19955

pED_50_^b^ = −LogED_50_

## DISCUSSION

Considering the hydrophobic contours of CoMSIA (Model 1), the yellow(Y) contours denote regions where hydrophobic groups are favored, while white (W) contours indicate regions where hydrophilic groups can be incorporated. Figure 6a shows that the 2-methoxyethyl substituent at the first position of the imidazole nucleus is embedded in a big yellow (Y) contour, indicating that the substitution with hydrophobic groups will increase the activity. The second position of the imidazole ring and the eighth position of the naphthalene ring are embedded in white (W) contour, indicating that introduction of hydrophilic groups at these positions will increase the activity. Similarly the third position of the napthyl group is surrounded by a white (W) contour indicating that addition of hydrophobic groups will decrease the activity. In the H-bond acceptor field [Figure 6b], magenta (M) contours represent the favored region, while red (R) contours show the disfavored region. The second position of the imidazole ring is embedded in big red (R) contour, indicating that substitution with the hydrogen bond donor group may increase the activity, while the fourth and fifth position of imidazole is embedded in large magenta (M) contours, indicating introduction of hydrogen bond acceptor groups may result in increased activity. The 2-methoxy group attached to the CH2CH2 side chain at the first position of the imidazole nucleus is embedded in a red (R) contour, indicating that activity may increase if this position is substituted with hydrogen bond donor groups.

## CONCLUSION

The CoMSIA analysis has been successfully applied to a series of 1-(Naphthylalky1)-1H –imidazole derivatives with anticonvulsant activity. The CoMSIA model (Model 1) was very well validated both internally and externally and proved to be the best of all the models developed. The robustness of the HA model was verified by the bootstrapping method. This model with a combination hydrophobic and H-bond acceptor fields (HA) indicates that hydrophobic and hydrogen bond acceptor groups may be important for the design of more potent imidazole analogs, as antiepileptic agents. Results of this study may be utilized for future drug design studies and synthesis of more potent antiepileptic agents with the arylalkylimidazole scaffold.
